# Protective Effects of Chitosan Oligosaccharide Against Lipopolysaccharide-Induced Inflammatory Response and Oxidative Stress in Bovine Mammary Epithelial Cells

**DOI:** 10.3390/md23010031

**Published:** 2025-01-09

**Authors:** Ziwei Lin, Yanlong Zhou, Ruiwen Chen, Qiuyan Tao, Qiwen Lu, Qianchao Xu, Haibin Yu, Ping Jiang, Zhihui Zhao

**Affiliations:** 1 The Key Laboratory of Animal Genetic Resource and Breeding Innovation, College of Coastal Agricultural Sciences, Guangdong Ocean University, Zhanjiang 524088, China; linzw@gdou.edu.cn (Z.L.); zyl72921@163.com (Y.Z.); w13822373092@163.com (R.C.); 15651760800@163.com (Q.T.); 18087276301@stu.gdou.edu.cn (Q.L.); 16603730974@163.com (Q.X.); yuhb@gdou.edu.cn (H.Y.); 2 The Key Laboratory of Animal Resources and Breed Innovation in Western Guangdong Province, Zhanjiang 524088, China

**Keywords:** chitosan oligosaccharide, lipopolysaccharide, TLR4/NF-κB signaling pathway, Nrf2 signaling pathway, bovine mammary epithelial cells

## Abstract

Chitosan oligosaccharide (COS) is receiving increasing attention as a feed additive in animal production. COS has a variety of biological functions, including anti-inflammatory and antioxidant activities. Mastitis is a major disease in dairy cows that has a significant impact on animal welfare and production. Hence, this research aimed to investigate the mechanism of COS on the lipopolysaccharide (LPS)-stimulated inflammatory response and oxidative stress in bovine mammary epithelial cells (BMECs). In this study, the results demonstrated that COS protected BMECs from the inflammatory response induced by LPS by restraining the excessive production of toll-like receptor 4 (TLR4), tumor necrosis factor-α (TNF-α), interleukin-6 (IL-6), and interleukin-1β (IL-1β). COS treatment also suppressed excessive reactive oxygen species (ROS) production and restored antioxidant enzyme activity under LPS-induced oxidative stress conditions. Furthermore, the results also demonstrated that COS promote nuclear factor erythroid 2-related factor 2 (Nrf2) expression and inhibit TLR4 levels and p65 and IκBα phosphorylation in BMECs exposed to LPS. In summary, the results demonstrate that the protective mechanism of COS on the LPS-induced inflammatory response and oxidative stress depend on the TLR4/nuclear factor-κB (NF-κB) and Nrf2 signaling pathways, indicating that COS could serve as natural protective agents for alleviating BMECs in mastitis.

## 1. Introduction

Dairy cows are crucial economic animals because they produce milk for human consumption. Therefore, ensuring the health of dairy cows can promote the development of the dairy industry. Dairy mastitis, a dairy cow disease with high incidence, seriously compromises the productivity, welfare, health, and fertility of dairy cows [1]. After suffering from mastitis, dairy cows lose a total of 110 to 552 kg throughout the entire lactation period [2]. Moreover, mastitis reduces the quality of milk, and prevents the sale of milk from cows affected by mastitis to consumers. In addition, dairy cows with mastitis also incur costs associated with treatment, including medicine and veterinary services. Occasionally, diseased dairy cows are culled [3]. Therefore, dairy mastitis leads to significant economic losses for the dairy industry [3]. Traditionally, mastitis can be controlled with antibiotic therapy, but this may be associated with significant side effects, such as antibiotic resistance [2]. In addition, antibiotic residues in milk also pose a health hazard to consumers. Therefore, dairy mastitis is also a food safety issue [4]. Currently, utilizing beneficial natural anti-inflammatory compounds with antioxidant properties may help overcome the problems associated with the use of traditional medicines as adjunctive treatments for mastitis. Therefore, screening for safe and effective candidate therapies from natural products to treat mastitis is important.

Chitosan oligosaccharide (COS) are degradation products prepared via the chemical or enzymatic hydrolysis of chitosan or chitin extracted from shrimp and crab shells [5]. They are oligomers that consist of β-(1-4)-linked D-glucosamine with 2 to 20 degrees of polymerization [6,7,8]. Moreover, due to their physicochemical properties, such as low viscosity, high water solubility, biocompatibility, and biodegradability, COS have been extensively used in numerous industries, including food, medicine, and agriculture, in recent years [8]. In particular, COS exhibit anti-inflammatory and antioxidative activity in different cells and animal models [9]. Hence, this research sought to investigate the effect of COS against inflammatory and oxidative stress induced by lipopolysaccharide (LPS) in a bovine mammary epithelial cell (BMEC) model that is commonly used to study the pathophysiology of mastitis in vitro [10].

BMECs, which are located on the apical surface of the mammary glands, are not only involved in the synthesis and secretion of milk but also make a vital contribution to mammary gland immune defense [11]. LPS is a major coliform endotoxin and it has a critical role in various inflammatory diseases, including mastitis [12]. It not only stimulates the inflammatory response but also causes oxidative stress in BMECs [13]. Thus, alleviating oxidative stress and inflammatory responses in BEMCs are recognized as important strategies for treating mastitis [12]. When released from bacteria, LPS binds to the toll-like receptor 4 (TLR4), which is a vital pattern-recognition receptor, resulting in inflammation. The nuclear factor-κB (NF-κB), the downstream signaling molecule of TLR4, is involved in inflammation related to bovine mastitis [14]. TLR4/NF-κB signaling pathway activation via LPS induces the transcription of pro-inflammatory cytokines including tumor necrosis factor-α (TNF-α), interleukin-1β (IL-1β), and interleukin-6 (IL-6). These cytokines conversely further aggravate the pathology and inflammatory responses of mastitis. Moreover, the Nrf2 signaling pathway is a suppressive regulator of oxidative stress induced by LPS [15]. Therefore, studying the effects of COS against LPS-challenged BMECs from the TLR4/NF-κB and Nrf2 pathways may reveal potential strategies against LPS-induced inflammatory and oxidative stress in BEMCs.

The objective of this research is to explore the antioxidative and anti-inflammatory functions of COS in BMECs stimulated with LPS. In addition, whether COS could alleviate the inflammatory response and oxidative stress via the TLR4/NF-κB and Nrf2 signaling pathways was also investigated. This research provides new insights for the potential application of marine drugs in animal husbandry.

## 2. Results

### 2.1. Effect of LPS and COS on BMEC Viability

**Figure 1:**
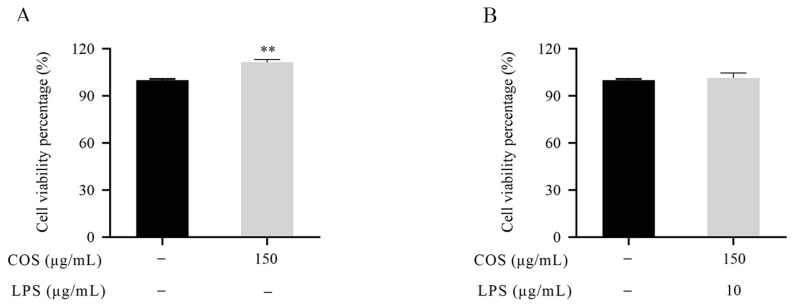
Effect of COS and LPS on BMEC viability. (**A**) Viability of BMECs treated with 150 μg/mL COS for 36 h. (**B**) Viability of BMECs pretreated with 150 μg/mL COS for 12 h and co-treated with 10 μg/mL LPS for an additional 24 h. Data are presented as the mean ± standard deviation (SD) (*n* = 3). ** *p* < 0.01 indicates a statistically significant difference.

As shown in Figure 1A, BMEC viability increased after treatment with 150 μg/mL COS for 36 h (*p* < 0.05). These results indicate that 150 μg/mL COS exhibits no cytotoxic effects on the BMECs at 36 h. Our research previously demonstrated that BMECs treated with 10 μg/mL LPS for 24 h induced inflammation but did not affect BMEC viability [16]. Therefore, this research sought to assess the viability of BMECs co-treated with LPS and COS. In Figure 1B, pretreatment of BMECs with 150 μg/mL COS for 12 h and co-treatment with LPS at 10 μg/mL for an additional 24 h did not affect BMEC viability (*p* > 0.05). Therefore, 10 μg/mL LPS and 150 μg/mL COS were used in the following experiments.

### 2.2. COS Alleviated Inflammatory Cytokine Secretion in LPS-Induced BMECs

**Figure 2:**
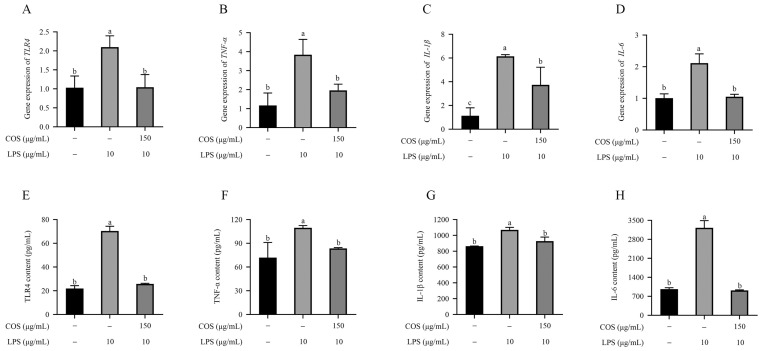
COS alleviated the LPS-induced inflammatory response in BMECs. The mRNA levels of (**A**) *TLR4*, (**B**) *TNF-α*, (**C**) *IL-1β*, and (**D**) *IL-6* were assessed using real-time quantitative PCR (RT-qPCR). The protein expression of (**E**) TLR4, (**F**) TNF-α, (**G**) IL-1β, and (**H**) IL-6 was tested using ELISAs. Data are presented as the mean ± SD (*n* = 3). Groups with different superscript letters were considered statistically different (*p* < 0.05)).

Compared with the control group, *TLR4*, *TNF-α*, *IL-1β*, and *IL-6* mRNA expression levels were significantly increased in BMECs treated with 10 μg/mL LPS (*p* < 0.05) (Figure 2A–D). In contrast, pretreatment with 150 μg/mL COS effectively significantly reversed this trend (*p* < 0.05). Consistently, in Figure 2E–H, the enzyme-linked immunosorbent assay (ELISA) results confirmed that COS pretreatment significantly decreased the expression of TLR4, TNF-α, IL-1β, and IL-6 induced by LPS (*p* < 0.05). The above findings suggested that COS potentially mitigate the inflammatory response by restraining inflammatory cytokines.

### 2.3. COS Mitigated Oxidative Stress in LPS Induced in BMECs

**Figure 3:**
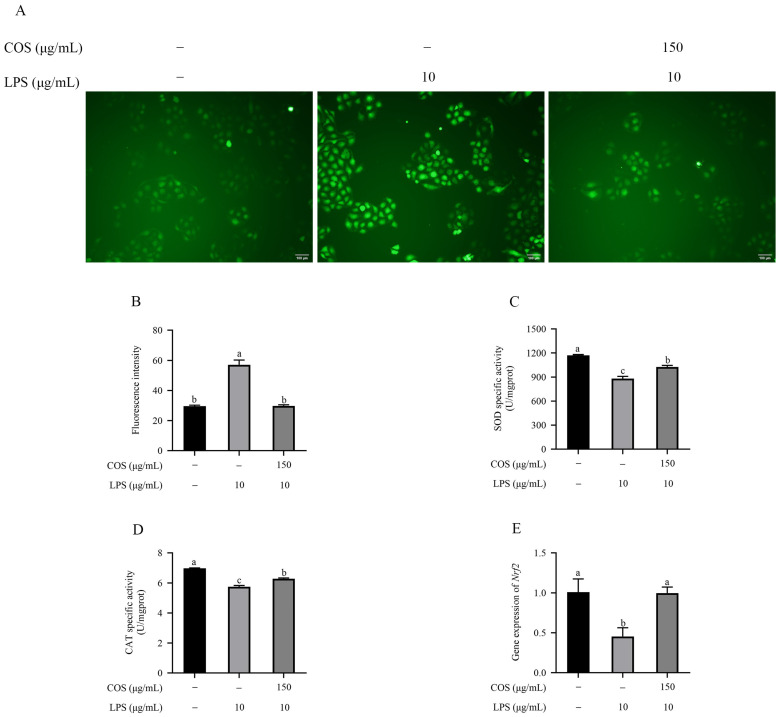
COS mitigated LPS-induced oxidative stress in BMECs. (**A**) Effects of COS on ROS fluorescence intensities in LPS-induced BMECs. Fluorescence microscopic images showing ROS production. (**B**) The fluorescence intensities were analyzed using Image J. (**C**,**D**) SOD and CAT activities. (**E**) *Nrf2* mRNA expression as assessed with RT-qPCR. Scale bar = 100 μm. Data are presented as the mean ± SD (*n* = 3). Groups with different superscript letters are statistically different (*p* < 0.05).

Reactive oxygen species (ROS) levels were assessed using an immunofluorescence microscopy and analyzed with Image J. In Figure 3A,B, the fluorescence intensity of the LPS group was significantly increased compared with the control group (*p* < 0.05). However, the fluorescence intensity of ROS in the COS with LPS group was markedly reduced compared to the LPS group (*p* < 0.05). Therefore, the results suggest that COS might protect BMECs against ROS oxidative damage.

Compared with the control group, LPS exposure prominently restrained superoxide dismutase (SOD) and catalase (CAT) activities, and COS pretreatment effectively reverses this phenomenon (*p* < 0.05) (Figure 3C,D). In addition, *Nrf2* mRNA expression in the control group was significantly lower that of the LPS group (*p* < 0.05) (Figure 3E). However, COS pretreatment markedly alleviated the reduction in Nrf2 expression induced by LPS (*p* < 0.05). The above results revealed that COSs relieve the oxidative stress induced by LPS in BMECs.

### 2.4. Effect of COS on Nrf2 and the TLR4/NF-κB Signaling Pathway in BMECs Stimulated with LPS

**Figure 4:**
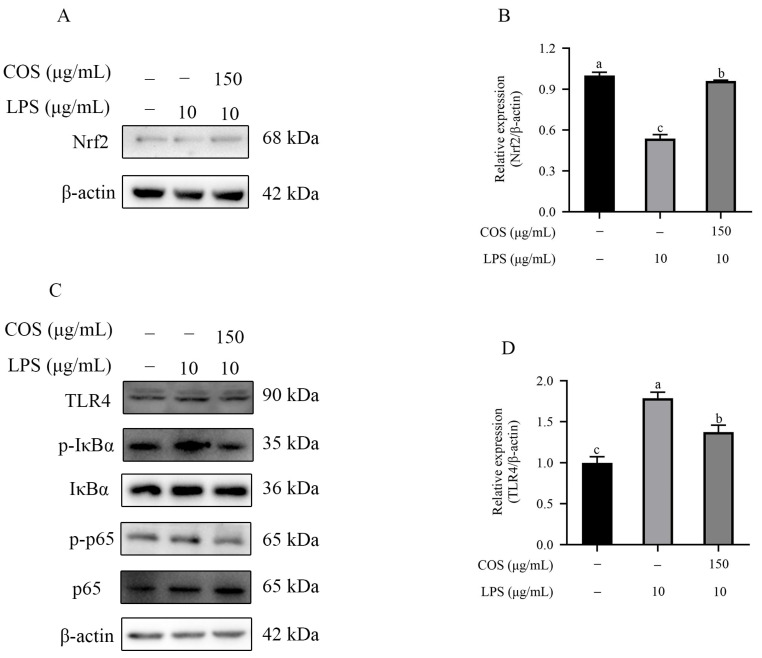

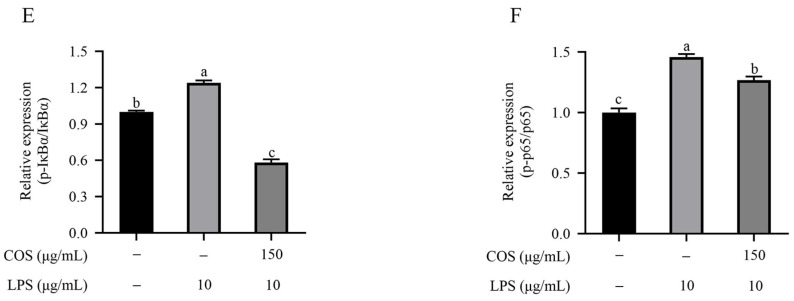
COS alleviated oxidative stress and inflammatory response induced by LPS by regulating Nrf2 and the TLR4/NF-κB signaling pathway. (**A**) Western blot (WB) images of Nrf2. (**B**) Quantification analysis of Nrf2 expression. (**C**) WB images of TLR4, p-IκBα, IκBα, p-p65, and p65. The quantification analysis of (**D**) TLR4, (**E**) p-IκBα/IκBα, and (**F**) p-p65/p65 expression. Data are presented as the mean ± SD (*n* = 3). Groups with different superscript letters are considered statistically different (*p* < 0.05).

In Figure 4A,B, Nrf2 expression in the LPS group markedly reduced compared with that of the control group. In contrast, Nrf2 expression was up-regulated in the COS with LPS group (*p* < 0.05). As demonstrated in Figure 4C–F, TLR4, p-IκBα/IκBα, and p-p65/p65 levels dramatically increased after LPS stimulation. However, TLR4, p-IκBα/IκBα, and p-p65/p65 levels in the COS with LPS group were reduced compared with the LPS group (*p* < 0.05). These results suggested that COS potentially alleviates oxidative stress and the inflammatory response stimulated by LPS in BMECs though Nrf2 and the TLR4/NF-κB signaling pathway.

## 3. Discussion

Cow mastitis restricts the healthy development of the dairy farming industry [17]. Therefore, mitigating inflammation and oxidative stress damage in cows with mastitis has become increasingly important [11]. Interestingly, COS represents a good source material against inflammatory responses and oxidative stress that has been widely used in animal models and clinical trials [8,9]. In this study, BMECs were treated with LPS to generate a mastitis model in vitro. The model was used to investigate the protective effects of COS on LPS-induced BMECs. We found that COS alleviate the inflammatory and oxidative responses induced by LPS in BMECs.

BMECs, the first line of defense in the cow’s mammary gland, release large amounts of cytokines to recruit and activate leukocytes to initiate an immune response against microbial infection and bacterial invasion [18,19]. However, an excessive inflammatory response may lead to cell damage, which must be regulated to prevent cell infection. BMECs exposed to LPS induce a rapid inflammatory response characterized by the increased production of pro-inflammatory cytokines that induce inflammatory injury [15]. Therefore, reducing the excessive production of inflammatory cytokines may be an effective strategy to treat mastitis. Recently, the anti-inflammatory function of COS has attracted growing attention. Qiao et al. [20] reported that COS reduced serum IL-1β and TNF-α levels in mice with sepsis induced by LPS. Guo et al. [21] demonstrated that COS restrained serum and colonic IL-1β, TNF-α, and IL-6 levels in mice treated with dextran sulfate sodium. In our research, TNF-α, IL-1β, and IL-6 levels increased when BMECs were stimulated with LPS. Similarly, COS significantly inhibited the production of TNF-α, IL-1β, and IL-6. The above findings suggest that COS potentially mitigate the inflammatory injuries induced by LPS in BMECs.

Moreover, an interaction occurs between the inflammatory response and oxidative stress to resist pathogen invasion [22]. COS exhibit excellent antioxidant properties both in vitro and in vivo [23]. Hence, after confirming the anti-inflammatory properties of COS in LPS-induced BMECs, we further evaluated whether COS alleviated oxidative stress stimulated by LPS. ROS are essential for vital physiological functions like cellular proliferation and differentiation, serving as an inflammatory mediator in inflammatory diseases, and ROS overproduction causes oxidative stress [24]. Under normal conditions, ROS production and clearance maintain a balance [25]. When this balance is disrupted, the excessive ROS will cause cell damage. The accumulation of ROS is an early event in the response of BMECs to LPS stimulation [12]. As potent free radical scavengers, COS suppresses intracellular ROS formation [26]. Our research also demonstrated this phenomenon. In our study, LPS increased ROS production, whereas COS pretreatment effectively down-regulated the release of ROS induced by LPS in BMECs. Additionally, studies have reported that the enhanced antioxidative activity of COS are related to SOD and CAT activities [27]. For example, Zhang et al. [28] reported that COS significantly inhibited ROS production and increased SOD activity in H_2_O_2_-induced SH-SY5Y cells via the Nrf2/ARE signaling pathway. Qiao et al. [20] reported that COS mitigated oxidative stress by increasing GHS and CAT levels and reducing MDA levels in mice with sepsis induced by LPS. Zhang et al. [29] also found that COS reduced ROS production and alleviated the reduction in SOD and CAT activities induced by doxorubicin in H9C2 cells. This study also found that COS pretreatment enhanced the activities of vital antioxidative enzymes (CAT and SOD) in LPS-induced BMECs. Therefore, the above results indicate that COS serve as antioxidants, effectively relieving oxidative stress by regulating ROS production and enhancing the antioxidative enzyme activities in LPS-induced BMECs.

In this study, the possible underlying molecular mechanisms of the anti-inflammatory and antioxidative properties of COS were also investigated. Recently, the mechanism of natural product for treating mastitis has focused on the TLR4/NF-κB signaling pathway. For example, Che et al. [30] revealed that allicin mitigates the inflammatory response induced by LPS in BMECs by regulating the TLR4/NF-κB signaling pathway. He et al. [31] demonstrated that baicalein alleviates the TLR4/NF-κB signaling pathway in the treatment of mastitis. Another study reported that TLR4/NF-κB is involved in the inflammatory response related to bovine mastitis [14]. In addition, Shi et al. [32] reported that COS alleviated inflammation stimulated by LPS in IPEC-J2 cells and mice though restraining TLR4/NF-κB signaling pathway. Li et al. [33] demonstrated that COS restrain the vascular endothelial inflammatory response induced by LPS by blocking the NF-κB signaling pathway. Huang et al. [34] found that COS down-regulate IL-6, TNF-α, p-NF-κB p65, IKKα/β, and IκB expression in LPS-challenged piglets. Hence, according to the above studies, this research aimed to explore the effect of COS on the TLR4/NF-κB signaling pathway in LPS-induced BMECs. TLR4, an LPS-specific sensor, is involved in inflammation and repair processes [35]. The binding of LPS and TLR4 activates various intracellular pro-inflammatory signaling cascades, particularly the NF-κB pathway. Several studies have reported that NF-κB is a meaningful transcription factor during the inflammatory response [36]. When the NF-κB pathway is activated, the nuclear transcription factor p65 dissociates from IκBα and enters the nucleus, which further causes the excessive accumulation of various pro-inflammatory factors [37]. To further explore the potential mechanism by which COS mitigates proinflammatory cytokine production, we examined TLR4 expression, p65 phosphorylation, and IκBα degradation in the TLR4/NF-κB signaling pathway. These results demonstrated that COS significantly suppressed TLR4 expression, IκBα degradation, and NF-κB p65 phosphorylation induced by LPS in BMECs, thereby alleviating inflammation. Thus, the results of this research revealed that COS potentially alleviate inflammatory damage by preventing TLR4/NF-κB signaling pathway activation in BMECs.

Studies have shown that the Nrf2 signaling pathway potentially protects against the oxidative stress of mastitis [11,38]. Hence, we also aimed to investigate the antioxidative activity of COS in LPS-induced BMECs. Nrf2, a transcription factor, plays a vital part in antioxidant response within cells [39]. Under conditions of oxidative stress, Nrf2 is activated, thereby controlling the antioxidant defense system [40]. Nrf2 also regulates genes encoding antioxidant activities, like SOD and CAT [41]. It was reported that the antioxidant potential of COS is related to antioxidative genes (SOD and CAT) through transcriptional activation of Nrf2 [42]. Hence, Nrf2 expression was assessed in this study. The results showed that Nrf2 levels were reduced in BMECs exposed to LPS. Moreover, COS pretreatment effectively increased Nrf2 expression in the COS with LPS group. The above results indicate that COSs potentially activate the Nrf2 signaling pathway to alleviate oxidative stress in LPS-induced BMECs.

Of note, a study has found increased Nrf2 expression after LPS stimulation, whereas COS extracted from Pacific white shrimp shell chitosan decreased Nrf2 expression in LPS-induced RAW 264.7 macrophages [43]. This phenomenon was in contrast to our research results. We hypothesize that one of the reasons might be related to the target animals or different tissues of animals. Lan et al. [27] revealed that different COS concentrations (0, 200, 400, and 800 mg/kg) have different effects on CAT, GSH-Px, and T-SOD activities in different regions of intestinal samples (the duodenum, jejunum and ileum) in broilers aged 1 to 14 days. In addition, the another reason might associated with the use of COS from different sources with different molecular weights, degrees of deacetylation, structures, and polymerization features, which might cause certain differences in their antioxidant and anti-inflammatory properties [27,44]. In the future, we will also compare COS with different characteristics to explore their differences in biological activity in BMECs. Therefore, when using COS in animal husbandry, breeders need to pay attention to their relevant characteristics.

## 4. Materials and Methods

### 4.1. BMECs Culture and Treatment

BMECs were obtained from the Laboratory of Molecular Genetics at Guangdong Ocean University. All experiments were performed according to the Guangdong Ocean University Ethics Committee. The BMECs were cultured using DMEM/F12 medium (Hyclone, Logan, UT, USA) and 10% fetal bovine serum (Zeta Life, Menlo Park, CA, USA). BMECs were incubated at 37 °C and 5% CO_2_.

COS (average molecular weight ≈ 1000, degree of polymerization 3–7, purity 97%) were purchased from Yuan Ye Biology (Shanghai, China). The COS lot number was F18IB207359, and the certificate of analysis for the COS is available in the Supplementary Materials. In addition, LPS (*Escherichia coli* O111:B4) was purchased from Sigma-Aldrich (St. Louis, MO, USA). Treatment with 150 μg/mL COS for 36 h did not affect BMEC viability [45]. Therefore, 150 μg/mL COS and 10 μg/mL LPS were used in the following experiments.

BMECs were separated into three groups: control group (without treatment), LPS group (BMECs treated with 10 μg/mL LPS for 24 h) and COS with LPS group (BMECs pretreated with 150 μg/mL COS for 12 h prior to exposure to 10 µg/mL LPS for 24 h).

### 4.2. Assessment of Cell Viability

Briefly, BMECs were cultured on 96-well plates at 5 × 10^3^ cells/well for 24 h. A Cell Counting Kit-8 (CCK-8) (Zeta Life, Menlo Park, CA, USA) assay was used to assess BMEC viability. According to the instructions, 10 μL CCK solution was added to per well of the plate, and the sample was incubated for 3 h. Subsequently, the absorbance was recorded at 579 nm using a microplate reader (BioTek, Winooski, VT, USA).

### 4.3. RT-qPCR

Total RNA was extracted from BMECs using Trizol reagent (Thermo Fisher Scientific, Waltham, MA, USA). The total RNA was reverse-transcribed into cDNA using a commercial kit (Vazyme, Nanjing, China). RT-qPCR was performed with the ChamQ Universal SYBR qPCR Master Mix (Vazyme, Nanjing, China) using the Applied Biosystems QuantStudio 1 system (Thermo Fisher Scientific, Waltham, MA, USA). The data were obtained using the 2^−^^∆∆Ct^ method. The primer sequences of the genes are provided in Table 1.

### 4.4. ROS Measurement

BMECs were cultured in six-well plates until the density of cells reached 80%. ROS levels were determined using Chekine^TM^ Reactive Oxygen Species Detection Fluorometric Assay Kit (KTB1910; Abbkine Scientific Co., Ltd., Wuhan, China). Briefly, the medium was replaced with 10 µmol/L 2′,7′-dichlorofluorescein diacetate (DCFH-DA), and samples were incubated for 30 min in the incubator. Subsequently, the DCFH-DA solution was removed, and BMECs were washed with a serum-free medium. Images were obtained using a microscope.

### 4.5. Antioxidant-Related Enzyme Activity Assays

SOD and CAT activities in BMECs were tested using corresponding commercial kits (SOD, E-BC-K020-M; CAT, E-BC-K031-M; Elabscience Biotechnology Co., Ltd., Wuhan, China). In brief, BMECs were homogenized in phosphate-buffered saline (PBS) (Hyclone, Logan, UT, USA) and centrifuged (1000× *g* and 4 °C) to collect the supernatant. A portion of the supernatant was used for protein concentration determination. The other portion was used to measure the SOD and CAT activities.

According to the manufacturer’s instructions, enzyme stock solution and enzyme diluent were mixed at a ratio of 1:10 to prepare the enzyme working fluid. Substrate solution and buffer solution were mixed at the ratio of 1:200 to prepare substrate application solution. Here, 20 μL of double diluted water and 20 μL of enzyme diluent were added to the blank well, and 20 μL of double diluted water and 20 μL of enzyme working liquid were added to the control well. In addition, 20 μL of the sample and 20 μL of enzyme working liquid were added to the test well. All wells received 200 μL of substrate application solution, and the plate was incubated at 37 °C for 20 min. Subsequently, the absorbance of SOD was recorded at 450 nm.

In addition, the CAT activities of different groups were measured as follows. Briefly, 20 μL of supernatant from different groups was mixed with 200 μL buffer solution. After incubating at 37 °C for 5 min, the substrate was added to the mixture, and the sample was incubated at 37 °C for 1 min. Then, 200 μL of chromogenic agent and 20 μL of clarifying agent were added to 20 μL of the test sample. The mixtures were incubated for 10 min. The absorbance of CAT was recorded at 405 nm.

### 4.6. ELISA

The supernatants of different groups were collected. TLR4, TNF-α, IL-1β, and IL-6 production were assessed using commercial detection kits (Jiangsu Meimian Industrial Co., Ltd., Yancheng, China) according to the manufacturer’s instructions. Briefly, 40 μL of sample diluent was added to 10 μL of the supernatant from different groups followed by 100 μL of horseradish peroxidase (HRP) conjugated detection antibody. The mixtures were incubated at 37 °C for 60 min. The liquid was discarded, and the well was washed with wash buffer five times. The plate was patted dry on absorbent paper. Subsequently, substrate A and B were added to each well, and the plate was incubated at 37 °C in the dark for 15 min. Finally, the stop solution was added, and the samples were measured at 450 nm.

### 4.7. Western Blot

The total proteins of BMECs from different groups were extracted using radio immunoprecipitation assay buffer (RIPA, Solarbio Science & Technology Co., Ltd., Beijing, China) with 1% protease and 1% phosphatase inhibitors (Solarbio Science & Technology Co., Ltd., Beijing, China), respectively. After quantitative extraction, proteins were separated by sodium dodecyl sulfate-polyacrylamide gel electrophoresis (SDS-PAGE). Subsequently, the protein was transferred onto polyvinylidene difluoride (PVDF) membranes (Millipore, Billerica, MA, USA). After blocking with 5% nonfat dried milk (Coolaber, Beijing, China), the membranes were incubated with primary antibodies at 4 °C overnight followed by the secondary antibody for 2 h at room temperature. The protein was visualized using a chemiluminescent system (Bio-Rad, Marnes-la-Coquette, France). Antibody information is listed in Table 2.

### 4.8. Statistical Analysis

Data are reported as the mean ± SD. The experimental data were assessed using GraphPad Prism version 8.4.0. Student’s *t*-test was used to analyze different groups with SPSS v23.0 software (IBM, Armonk, NY, USA). Protein bands were analyzed using Image J software v1.8.0 (Wayne Rasband, National Institutes of Health, Bethesda, MD, USA). *p* < 0.05 indicates a statistically significant difference.

## 5. Conclusions

**Figure 5:**
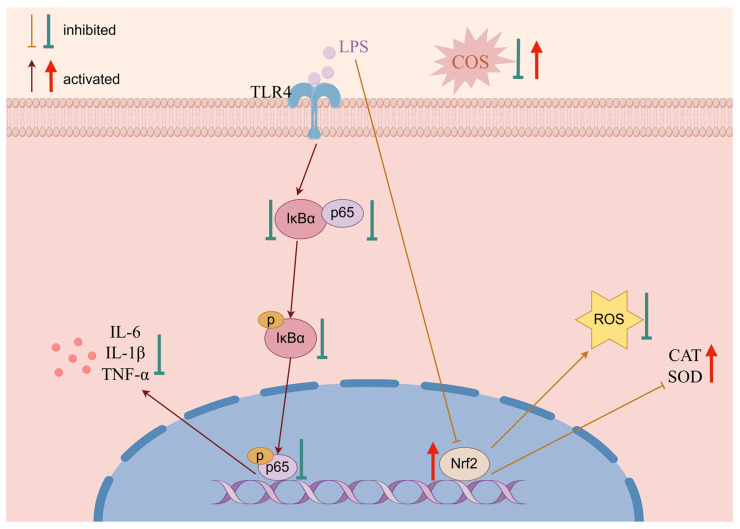
Protective mechanisms of COS in LPS-induced BMECs (image generated using Figdraw).

This research demonstrates that COS inhibited TLR4 expression and IκBα and p65 phosphorylation, thereby preventing the secretion of downstream inflammatory cytokines, such as TNF-α, IL-1β, and IL-6, in LPS-induced BMECs. COS decreased ROS production and increased SOD and CAT content in BMECs stimulated by LPS by activating the Nrf2 signaling pathway. In summary, COS potentially regulate the TLR4/NF-*κ*B and Nrf2 signaling pathway to alleviate the inflammatory response and oxidative stress, respectively (Figure 5).

## Figures and Tables

**Table 1:** Primer sequences used for RT-qPCR.

Genes	Sequence Number	Primer Sequences (from 5′ to 3′)
*TLR4*	NM_174198.6	F: GCAGGGAAAGTCAACTAAAC
		R: ACATAAAGTGGAGGGGAATC
*TNF-α*	XM_005223596.4	F: ACGGGCTTTACCTCATCTACTC
		R: TGGCAGACAGGATGTTGACC
*IL-1β*	NM_174093.1	F: GATGGCTTACTACAGTGACGA
		R: AGATGAATGAAAGGATGCTC
*IL-6*	NM_173923.2	F: GATGCTTCCAATCTGGGTTCA
		R: TCCTGATTTCCCTCATACTCG
*Nrf2*	NM_001011678.2	F: GTCTTCACTGCTCCTCCTCAG
		R: CTCCCAAACTTGCTCAATGTC
*β-actin*	NM_173979.3	F: AGAGCAAGAGAGGCATCC
		R: TCGTTGTAGAAGGTGTGGT

**Table 2:** Antibody information for Western blot.

Antibody	Catalog Number	Manufacturer
Nrf2	AF0639	Affinity Biosciences (Cincinnati, OH, USA)
TLR4	bs-20595R	Bioss (Beijing, China)
p65	10745-1-AP	Proteintech Group, Inc. (Wuhan, China)
p-p65	3033T	Cell Signaling Technology, Inc. (Danvers, MA, USA)
p-IκBα	bs-2513R	Bioss (Beijing, China)
IκBα	10268-1-AP	Proteintech Group, Inc. (Wuhan, China)
β-actin	AP0060	Bioworld Biotech Co., Ltd. (Nanjing, China)

## Data Availability

The data presented in this study are available on request from the corresponding author.

## Supplementary Materials

The certificate of analysis for the COS can be downloaded at: https://www.mdpi.com/article/10.3390/md23010031/s1.
