# Supplementary material for: Protective Effects of Chitosan Oligosaccharide Against Lipopolysaccharide-Induced Inflammatory Response and Oxidative Stress in Bovine Mammary Epithelial Cells
Source: Mar Drugs. 2025 Jan 9;23(1):31. doi: 10.3390/md23010031 (PMC11767086; doi:10.3390/md23010031)
Supplement: Supplementary file 1 [file marinedrugs-23-00031-s001.zip › marinedrugs-3388207-supplementary.pdf]

上海源叶生物科技有限公司
Shanghai yuanye Bio-Technology Co., Ltd
电话：400-666-5481　传真：021-55068248
网址：www.shyuanye.com
邮箱：3008007435@qq.com

质量检验报告
CERTIFICATE OF ANALYSIS

产品名称(Product Name)：壳寡糖

英文名称(English Name) ：Chitosan oligosaccharide

CAS：148411-57-8

储存条件(Storage Condition)：2-8℃

货号(Item No)：B25987

批号 (Lot. Number)：F18IB207359

检测日期(Date of Testing)：2023-02-18

复测日期(Date of Retesting)：2026-02-17

分析项目<br>SPECIFICATION PROPERTIES	技术指标<br>STANDARD	实测结果<br>RESULTS
Appearance	Faint yellow to yellow powder	Faint yellow powder
Assay	97%min	Conforms
Solubility	10mg/mL, H_2O	Clear，light-yellow solution
PH	5.5-7.0	5.6
Mol wt	≈ 1000	Conforms
Water	10.0%max	6.57%

上海源叶生物科技有限公司
质检专用章

Quality Assurance：Xiaoyong Tan

Quality Control：Yunfei Zhu
